# Production and Biochemical Characterization of a High Maltotetraose (G4) Producing Amylase from *Pseudomonas stutzeri* AS22

**DOI:** 10.1155/2014/156438

**Published:** 2014-05-26

**Authors:** Hana Maalej, Hanen Ben Ayed, Olfa Ghorbel-Bellaaj, Moncef Nasri, Noomen Hmidet

**Affiliations:** Laboratoire de Génie Enzymatique et de Microbiologie, Ecole Nationale d'Ingénieurs de Sfax, BP 1173, 3038 Sfax, Tunisia

## Abstract

Amylase production and biochemical characterization of the crude enzyme preparation from *Pseudomonas stutzeri* AS22 were evaluated. The highest **α**-amylase production was achieved after 24 hours of incubation in a culture medium containing 10 g/L potato starch and 5 g/L yeast extract, with initial pH 8.0 at 30°C under continuous agitation at 200 rpm. The optimum temperature and pH for the crude **α**-amylase activity were 60°C and 8.0, respectively. The effect of different salts was evaluated and it was found that both **α**-amylase production and activity were Ca^2+^-dependent. The amylolytic preparation was found to catalyze exceptionally the formation of very high levels of maltotetraose from starch (98%, w/w) in the complete absence of glucose since the initial stages of starch hydrolysis (15 min) and hence would have a potential application in the manufacturing of maltotetraose syrups.

## 1. Introduction

Maltotetraose, known as G4, is an oligosaccharide of 4 units of *α*-D-glucopyranose linked by *α*-(1–4) bond. This compound has a considerable specific interest like its use as a substrate of amylases to study their mode of action and as a highly sensitive substrate for detection of specific *α*-amylase activity when coupled with a chromogenic compound [1]; it also finds potential applications in the food industries due to its properties [2, 3]. In fact, it can be used in baking due to its high moisture retention power which serves to prevent retrogradation of starch ingredient [4]. Maltotetraose is also being tested for its use as a food additive to improve the texture or to reduce the sweetness of foods without affecting their inherent taste and flavor [5, 6].

Besides the above properties, G4 syrup, considered as a partially undigested and unabsorbed substrate in the small intestine, has shown a prebiotic effect by selectively promoting the growth and/or activity of beneficial bacteria, once it reaches the colon. In fact it has been demonstrated through* in vitro* and* in vivo* experiments that oligosaccharides were utilized by bifidobacteria classified as beneficial intestinal bacteria, but they were not utilized by* Escherichia coli* or the* Clostridium* species which were unfavourable for their producing putrefactive substances (protein degrading) in the digestive tract. Consequently, the ingestion of G4 syrup could improve intestinal flora and suppress the formation of putrefactive products [7].

However, the preparation of maltooligosaccharides with a specific degree of polymerisation (DP) in larger amounts is so expensive. Therefore, the discovery of microbial enzymes that produce from starch maltooligosaccharides of a specific length has made it possible to produce with good yield various maltooligosaccharides [8].

Microbial amylases are produced mainly from cultures of* Aspergillus*,* Bacillus*,* Streptomyces*, and* Pseudomonas* species [9]. Amylolytic activity is one of phenotypic characteristics of* Pseudomonas stutzeri* species [10], especially G4-*α*-amylases that have been subject of intense biochemical research such as purification and biochemical characterization [11].

In the view of advantages offered by the use of the amylolytic preparation instead of the purified enzyme, such as to avoid enzyme purification procedures that are expensive and time-consuming, we report in the present study the isolation and characterization of a novel G4-*α*-amylase producing bacteria* Pseudomonas stutzeri* AS22 from Tunisian soil samples. Conditions of *α*-amylase production were optimized to achieve high enzyme production and the amylolytic preparation was characterized.

## 2. Materials and Methods

### 2.1. Strain Isolation and Identification

**Figure 1:**
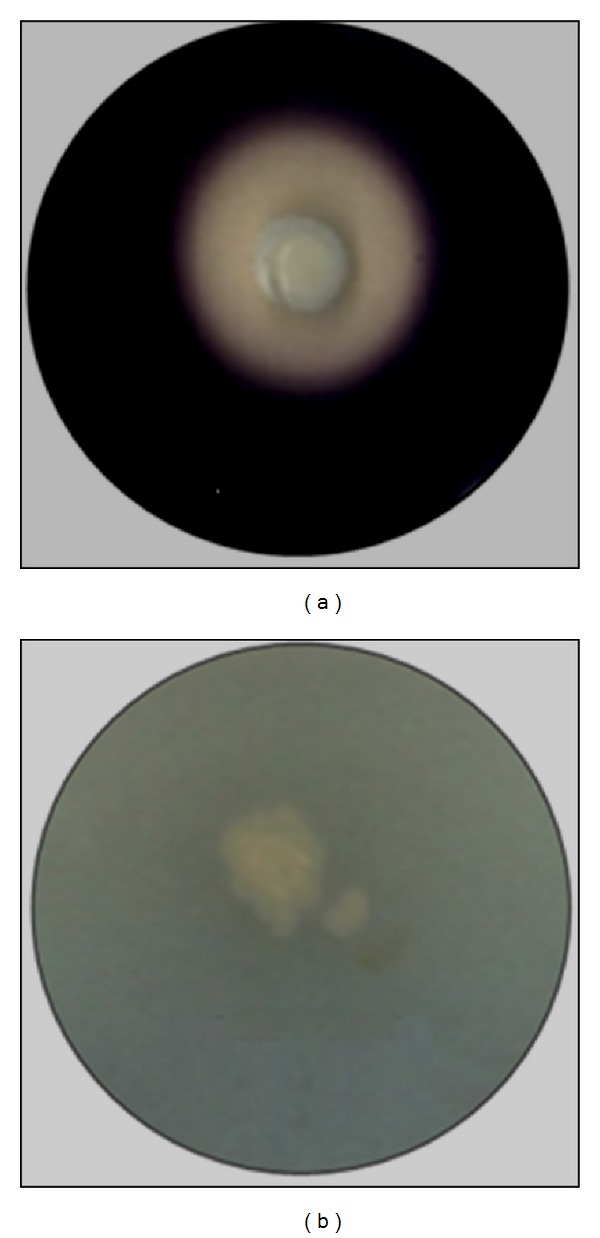
Amylolytic (a) and proteolytic (b) activities of the isolated strain AS22. The amylolytic and proteolytic activities of the strain AS22 were evaluated by puncture inoculation of the strain inside nutrient agar medium containing soluble starch (1% w/v) (a) and casein (b), respectively. After incubation at 37°C for 24 h, to detect the amylolytic activity, the plates were flooded with iodine solution at room temperature.

Various soil samples were collected in the region of Sfax (Tunisia) and different microorganisms were screened for their amylolytic activity using nutrient agar plates containing soluble starch (1% w/v) (Figure 1(a)). The strain, having the highest activity, was identified as* Pseudomonas stutzeri* by using the phylogenetic analysis based on the 16S rDNA sequence analysis. Genomic DNA, for the PCR template, was isolated from bacterial cells grown in Luria-Bertani (LB) media overnight by the* Wizard Genomic DNA Purification Kit* from Promega and amplified using the universal oligonucleotide primers (Bio Basic Inc.) 16SF (5′GCTAACTAACGTGCCAGCAG) and 16SR (5′CCCGGGATCCAAGCTTAAGGAGGTGATCCAGCC). Nucleotide sequence of the amplified 16S rDNA gene region was compared with those available in the GenBank database by using the BLAST method. The BLAST result showed that the 16S rDNA sequence of the isolated strain AS22 has 99% sequence similarity with the strain* Pseudomonas stutzeri*.

### 2.2. Medium Composition and Culture Condition

Inocula were routinely grown in Luria-Bertani (LB) broth medium composed of (g/L) peptone 10.0, yeast extract 5.0, and NaCl 5.0 [12], and the initial pH was adjusted to 7.0.

The basal liquid culture medium used for *α*-amylase production by the* P. stutzeri* AS22 strain was composed of (g/L) carbon source 10, ammonium sulphate as nitrogen source 1, MgSO_4_ (7 H_2_O) 0.1, K_2_HPO_4_ 1.4, KH_2_PO_4_ 0.7, and NaCl 0.5. The medium was adjusted to pH 8.0. Media were autoclaved at 121°C for 20 min.

The strain was cultivated in 250 mL conical flasks containing 25 mL medium inoculated at initial OD of 0.016 and maintained for 24 h at 37°C and 200 rpm. The cultures were centrifuged at 13.000 rpm for 15 min, and the cell-free supernatants were evaluated for their amylolytic activity.

### 2.3. *α*-Amylase Activity Assay

*α*-Amylase activity was measured by the determination of reducing sugars released during starch hydrolysis, by the dinitrosalicylic acid (DNS) method [13]. The reaction mixture, containing 0.5 mL of appropriately diluted enzyme and 0.5 mL of 1.0% (w/v) soluble potato starch (Sigma) in 100 mM Tris-HCl buffer (pH 8.0), was incubated at 60°C for 10 min. After that, 3 mL of DNS reagent was added to the reaction volume, boiled for 10 min, and mixed with 20 mL distilled water. To determine the activity, the absorbance was measured at 550 nm and one unit (U) of *α*-amylase activity was defined as the amount of enzyme that released 1 *μ*mol of reducing end groups per minute under the assay conditions.

### 2.4. *α*-Amylase Localization

The 24 h culture broth (25 mL) of* P. stutzeri* AS22 was centrifuged at 13.000 ×g for 10 min, and the supernatant was considered as the extracellular fraction. The cell pellet was washed twice with distilled water and suspended in 5 mL of Sodium Chloride-Tris-EDTA (STE) buffer containing 10 mM Tris-HCl (pH 8.0), 100 mM NaCl, and 1 mM EDTA. Then, lysozyme was added to a final concentration of 200 *μ*g/mL and the mixture was incubated at 0°C for 1 h. After centrifugation at 8.000 ×g for 25 min, the supernatant was considered as the periplasmic fraction and 5 mL of Tris-HCl buffer (100 mM, pH 8.0) was added. The obtained mixture was sonicated twice for 3 min each time to disrupt cells. The homogenate was finally centrifuged, and its supernatant was considered as the intracellular fraction. Finally, the obtained three fractions were tested for their amylolytic activity.

### 2.5. Optimization of *α*-Amylase Production

#### 2.5.1. Effects of Different Carbon Sources

The effects of different carbon sources (glucose, lactose, maltose, potato starch, wheat starch, and maize starch) on *α*-amylase production by the* P. stutzeri* strain were examined at a concentration of 1%, keeping constant the rest of the media composition. The best of these carbon sources was further optimized in the range of 0.25–2% (w/v).

#### 2.5.2. Effects of Different Nitrogen Sources

To investigate the effects of different nitrogen sources on *α*-amylase production, ammonium sulphate in the basal medium, containing 10 g/L potato starch, was replaced with different organic (yeast extract, casein, pastone, and soya peptone) and inorganic (ammonium sulphate and ammonium chloride) compounds as nitrogen source at a concentration of 0.1% (w/v), keeping constant the rest of the media composition. The concentration of the selected nitrogen source was further optimized in the range of 0.1–1% (w/v).

#### 2.5.3. Effects of Temperature, Agitation, Initial pH, Salts, and Incubation Time on *α*-Amylase Production

The effect of incubation temperature on *α*-amylase production was investigated by incubating the media for 24 h at different temperatures (25, 30, 37, and 45°C) in an automatic incubator.

The effect of agitation on enzyme production was also determined by incubating the inoculated culture flasks in an automatic mechanical shaker for 24 hours at 150, 200, and 250 rpm and then checking for extracellular *α*-amylase production.

To investigate the effect of pH on enzyme production, the initial pH of the medium was adjusted from 6.0 to 12.0.

KH_2_PO_4_ (0.7 g/L), K_2_HPO_4_ (1.4 g/L), NaCl (0.5 g/L), MgSO_4_, and CaCl_2_ were incorporated into growth medium to study the effects of mineral sources on *α*-amylase production. The basal medium containing 10 and 5 g/L potato starch and yeast extract, respectively, was used as control. To study the effect of CaCl_2_ and MgSO_4_, these chemicals were added to the medium so that their final concentration ranged from 0.1 to 0.8 g/L. Amylolytic activity was determined after incubation for 24 h at 30°C and 200 rpm agitation speed.

For maximal *α*-amylase production, fermentation period (0–240 h) was also optimized under the optimized fermentation conditions. Fermentation was performed at 30°C in an automatic incubator and samples were prepared at different intervals (continuously for 10 days) for enzyme assay.

### 2.6. Biochemical Properties of the Crude *α*-Amylase Preparation

#### 2.6.1. Effect of Temperature on Activity and Stability

The effect of temperature on *α*-amylase activity was studied from 20 to 90°C. Thermal stability was examined by incubating the enzyme preparation for 60 min at different temperatures ranging from 30 to 70°C. Aliquots were withdrawn at desired time intervals and the remaining activity was measured under enzyme assay conditions. The nonheated enzyme was taken as 100%.

#### 2.6.2. Effect of pH on Activity and Stability

The effect of pH on *α*-amylase activity was evaluated for the pH range of 3.0–13.0 at 60°C. For pH stability measurement, the enzyme was incubated at 30°C for 1 h in different buffers and the residual activity was determined under the enzyme assay conditions. The following buffer systems were used: 100 mM glycine-HCl buffer, pH 3.0-4.0; 100 mM acetate buffer, pH 4.0–6.0; 100 mM Tris-HCl buffer, pH 7.0-8.0; 100 mM glycine-NaOH buffer, pH 9.0–11.0; and 100 mM Na_2_HPO_4_-NaOH buffer, pH 12.0 and 13.0.

#### 2.6.3. Effect of Metal Ions, Surfactants, and Enzyme Inhibitors

The influence of various metal ions (5 mM) on *α*-amylase activity was investigated using CaCl_2_, ZnCl_2_, FeCl_2_, HgCl_2_, BaCl_2_, MnCl_2_, MgCl_2_, CuCl_2_, CdCl_2_, NaCl, and KCl. Activity in the absence of any additives was taken as 100%.

The effects of some surfactants (Triton X-100, Tween 20, Tween 80, and SDS) and enzyme inhibitors (phenylmethylsulfonyl fluoride (PMSF), *β*-mercaptoethanol, and ethylene-diaminetetraacetic acid (EDTA)) on *α*-amylase stability were studied by preincubating the amylolytic preparation for 30 min at room temperature. The remaining enzyme activity was measured under enzyme assay conditions. The activity of enzyme incubated under similar conditions without any additive was taken as 100%.

### 2.7. Analysis of End Products Starch Hydrolysis

Starch solution (1% w/v, 50 mL) was prepared using Tris-HCl buffer (100 mM, pH 8.0). The* P. stutzeri* AS22 crude *α*-amylase (0.6 U) was added into 50 mL of starch solution. Reaction mixture was incubated at 60°C. Aliquots were withdrawn at various time intervals and boiled for 10 min to stop the reaction and samples were then centrifuged at 13.000 g for 10 min and passed through 0.22 *μ*m filter. Samples thus obtained were analyzed by thin-layer chromatography (TLC) and gel permeation chromatography (GPC) on Bio-Gel P2.

TLC was performed on silica gel 60 (20 × 20 cm, Merck, Germany) with a mobile phase composed of chloroform/acetic acid/water (60 : 70 : 10, v/v/v). The spots were visualized by spraying TLC plates with H_2_SO_4_/ethanol (5 : 95, v/v) followed by heating at 120°C for 10 min.

GPC was performed on a Bio-Gel P2 column (1.5 × 200 cm) eluted with water at a rate of 30 mL/h. The different oligosaccharides in the range of G1 to G7 were fractionated and the percentages of different products were determined.

### 2.8. Visualisation of *α*-Amylase Activity by Zymography

*α*-Amylase activity staining was done by layering the SDS-PAGE gel on a thin 2% agarose-1% soluble potato starch gel incubated as a sandwich for 60 min at 50°C. Upon staining the agarose gel with iodine solution at room temperature, protein bands with amylolytic activity became visible as a white band against a dark blue background.

## 3. Results and Discussion

### 3.1. *α*-Amylase Production by* P. stutzeri* AS22

The production of amylases enzymes by microorganisms is significantly affected by physical and chemical parameters of the medium [14, 15]. In this regard, appropriate media components and suitable conditions must be attained for optimal production of the required products.

#### 3.1.1. Effects of Different Carbon Sources on *α*-Amylase Production

Because amylase synthesis is known to be induced by starch or its hydrolytic products [16–18],* P. stutzeri *AS22 was grown in the basal medium supplemented with starch from various natural sources (potato, wheat, and maize starch) and other carbohydrates including lactose, maltose, and glucose, for assessing their effects on the production of amylase (Table 1).

The highest amylase activity (0.8 U/mL) was produced on potato starch followed by maltose (0.5 U/mL). However, amylase production was significantly low when the strain was grown on lactose (0.055 U/mL) and glucose (0.1 U/mL). This study falls in line with previous studies on* P. stutzeri* amylase production, in which enzyme was induced using starch, amylodextrin or maltose, while glucose was found to inhibit amylase production [1, 19–21]. In contrast to our results, glucose was found to be the best carbon source for amylase production by* Pseudomonas* sp. IMD 353 (13 U/mL), while the amylolytic activity decreased to 2 and 3 U/mL, when maltose and starch were used, respectively, as sole carbon sources in the same conditions [22].

Since potato starch was the best carbon source for amylase synthesis, the effect of its concentration (0.25–2%) on the amylase production was studied in media containing 0.1% ammonium sulphate as nitrogen source. It was observed that the increase in concentration of potato starch increases amylase production and maximum activity (0.75 U/mL) was obtained in the presence of 1% substrate (data not shown). However, further increase (1.5 and 2%) of potato starch concentration resulted in rapid decrease of enzyme production although biomass remained nearly constant (decreased slightly). This may be explained by the degradation, during the fermentation, of starch by *α*-amylases, resulting in the accumulation of high quantities of reducing sugar, which led to an enhancement of sugar concentration and therefore to catabolite repression of *α*-amylase synthesis [17].

#### 3.1.2. Effects of Various Nitrogen Sources on *α*-Amylase Production

In general, both organic and inorganic nitrogen sources were used efficiently in the growth medium for the biosynthesis of *α*-amylase.

In the present study, various nitrogen sources at a concentration of 0.1% were evaluated using an optimum potato starch concentration of 1% (Table 2). Maximum activity was obtained when yeast extract was used (2.5 U/mL). Addition of yeast extract to the medium increased the production of *α*-amylase activity by more than three times over medium with ammonium sulphate as nitrogen source. Compared to the organic nitrogen sources which support good growth and extracellular *α*-amylase production, inorganic nitrogen sources like ammonium sulphate and ammonium chloride are not efficient for amylase production. These results corroborate well with previous studies showing that organic nitrogen sources were preferred for the **α**-amylase production. The stimulatory effect of these complex compounds may be attributed to their carbohydrate and protein compositions, as well as to the trace of minerals and ions that could be present and which enhance the enzyme secretion [18]. Yeast extract has been also used as nitrogen source in the culture medium for amylase production, alone in the case of* P. stutzeri* NRRL B-3389 [19], or in combination with other nitrogen sources such as polypeptone in the case of* Pseudomonas* strain MS300 [23] and yeatex for* Pseudomonas* sp. IMD 353 [22]. Other organic nitrogen sources have been also reported to support *α*-amylase production by* P. stutzeri *MO-19 such as corn steep liquor and peptone [20] and polypeptone in the case of* P. saccharophila* IAM 1504 [24].

To investigate the best concentration on *α*-amylase production, yeast extract (range 1–10 g/L) was added to basal medium containing potato starch (10 g/L). Maximum amylase production by* P. stutzeri *AS22 was obtained with 5 g/L yeast extract, reaching 3.95 U/mL and further addition of yeast extract decreased the level of amylase (data not shown).

#### 3.1.3. Effects of Temperature, Agitation, Initial pH Values, and Salts on *α*-Amylase Production

The effects of temperature (25 to 45°C), agitation (150, 200 and 250 rpm) and initial pH (6.0 to 12.0) on *α*-amylase production by* P. stutzeri* AS22 were studied in optimized medium containing 10 g/L potato starch and 5 g/L yeast extract.

Optimum level of *α*-amylase production (6 U/mL) was achieved at 30°C (data not shown). This result falls in line with previous studies on* Pseudomonas α*-amylases, in which most of them are reported to be produced at 25 [23] and 30°C [20, 24]. Amylase production seems to be very sensitive to higher temperatures. Indeed, at 37°C, the amylolytic activity was 40% lower than that at 30°C and at 45°C; no *α*-amylase activity was detected.

Among the tested agitation speeds, the maximum amylase activity (6.1 U/mL) was found at 200 rpm (data not shown).

The pH of the production medium strongly affects many enzymatic processes and transport of compounds across the cell membrane [18, 25]. In this study, the effect of pH on *α*-amylase production by* P. stutzeri *AS22 was investigated with varying pHs (6.0–12.0). The results revealed that the pH of the medium influences the *α*-amylase production as well as the strain growth (Table 3), which were more favorable at alkaline pH 8.0–12.0 than the neutral one. In contrast, acidic conditions (pH 6.0) seem to affect the *α*-amylase production, even when the stain might be able to grow. From this result, it has been understood that amylase production may occur at pH 6.0, but since the final pH was lowered to 4.5, no amylase activity was detected after 24 h of incubation because of the instability of the enzyme toward acidic conditions. Most of the earlier studies revealed neutral or alkaline initial pH for *α*-amylase production by* Pseudomonas* species [20, 22, 24]. On the other hand, Lalucat et al. [10] reported that none of the* Pseudomonas stutzeri *strains tolerate acidic conditions and they do not grow at pH 4.5.

In an attempt to increase the *α*-amylase production by* P. stutzeri*, the effect of some salts including K_2_HPO_4_ (1.4 g/L), KH_2_PO_4_ (0.7 g/L), NaCl (0.5 g/L), MgSO_4_ (0.1 g/L), and CaCl_2_ (0.1 g/L) was investigated. Results (Table 4) revealed that even though the growth rate was lower in medium supplemented with phosphate ions (as K_2_HPO_4_ and KH_2_PO_4_), production of *α*-amylase was enhanced as compared to the medium without salts addition (none). It is also interesting to note that while amylase production was slightly affected by the addition in the culture medium of the monovalent cation Na^+^ (in the form of NaCl), divalent cations (0.1 g/L) seem to enhance moderately or effectively the amylase production by* P. stutzeri* AS22, in the case of Mg^2+^ and Ca^2+^, respectively. As mentioned by Gupta et al. [18], differences in the mineral requirements of amylase producing bacteria have been reported and whereas most of previous studies indicated that addition of CaCl_2_ to the fermentation medium is known to be essential for amylase production [9, 26, 27], Ca-independent amylase production has also been reported [28, 29]. In comparison with the control medium, when CaCl_2_ (0.4 g/L) was added individually as a mineral source to the medium, amylase production was enhanced from 5.9 to 6.6 U/mL. However, by increasing the CaCl_2_ concentration from 0.8 to 1.2 g/L, decline in enzyme production by approximately 30% was observed. This finding is in accordance with previous studies showing inhibition of amylase production by the excess of some minerals such Ca^2+^ [30] and Mg^2+^ ions [31].

Maximum amylase production (6.8 U/mL) occurred when CaCl_2_ (0.4 g/L) was supplemented to the control medium containing potato starch 10 g/L, yeast extract 5 g/L, MgSO_4_ (7 H_2_O) 0.1 g/L, K_2_HPO_4_ 1.4 g/L, KH_2_PO_4_ 0.7 g/L, and NaCl 0.5 g/L.

#### 3.1.4. Pattern of *α*-Amylase Production by* P. stutzeri* AS22

**Figure 2:**
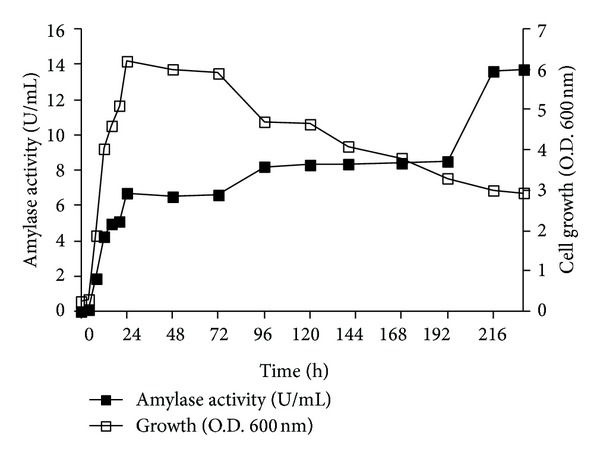
Pattern of growth and *α*-amylase production of* P. stutzeri* AS22 strain. Culture was conducted in media consisting of (g/L) potato starch 10, yeast extract 5, MgSO_4_ 0.1, K_2_HPO_4_ 1.4, KH_2_PO_4_ 0.7, CaCl_2_ 0.4 g/L, and NaCl 0.5 g/L. Incubation was carried out at 30°C, in a rotary shaker, with stirring at 200 rpm.

*P. stutzeri *AS22 was grown aerobically in the optimized medium (potato starch 10 g/L, yeast extract 5 g/L, MgSO_4_ (7 H_2_O) 0.1 g/L, K_2_HPO_4_ 1.4 g/L, KH_2_PO_4_ 0.7 g/L, and NaCl 0.5 and CaCl_2_ 0.4 g/L) for 240 h at 30°C. The pattern of *α*-amylase production and bacterial growth was followed with time** (** Figure 2). Amylase production was initiated during the exponential growth phase of the strain, reached 6.75 U/mL during the stationary phase at 24 h, and remained constant till 72 h, suggesting that the enzyme production is growth associated and induced by the presence of starch hydrolysis products in the medium. These results are in agreement with the reports of Nakada et al. [20] and Fogarty et al. [22] on the relationship between pattern of cell growth and *α*-amylase production.

However, the level of *α*-amylase increased slightly from 72 h to 96 h, and thereafter it increased more rapidly from 192 to 216 h, as the bacterial cells approached their death phase. Since the increase of *α*-amylase activity was concomitant with the cell optical density decrease, the amylase activity increase may be the result of bacterial cell dying and release of intracellular or periplasmic *α*-amylases in the culture medium. Therefore, attempts to confirm this statement resulted in the localization of *α*-amylase activity after 24 h of incubation. It was found that* P. Stutzeri* AS22 produced a dominant extracellular *α*-amylase as well as intracellular and periplasmic *α*-amylases, which constitute approximately 10% and 3% of the extracellular fraction, respectively.

**Figure 3:**
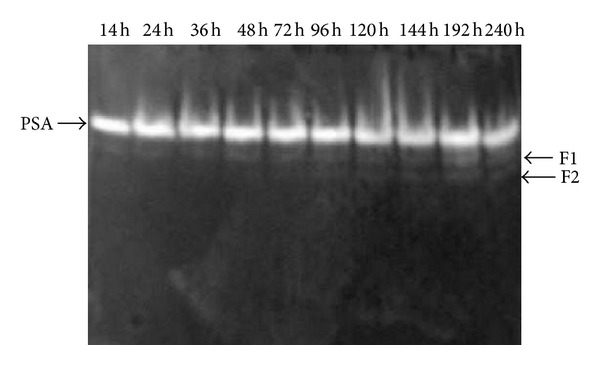
Changes in zymogram pattern for* P. stutzeriα*-amylases during cultivation.

A zymogram prepared using the cell-free culture supernatant at various time intervals (Figure 3) showed only one dominant protein band (PSA) in all the culture supernatants. Two minor amylases were also observed; one active form (F1) was detected in 14–240 hr culture supernatants and the other form (F2) was observed in 48–240 hr culture supernatants. The change of PAGE pattern led us to suspect that F1 and F2 are either produced from proteolytic degradation of PSA, or later secreted in the extracellular medium. However, since* P. stutzeri* AS22 lacks proteolytic activity (Figure 1(b)) and the intensity of the PSA band remains constant from beginning to 240 h, this clearly indicates that F1 and F2 are not derived from PSA proteolysis but are later secreted in the extracellular medium.

Multiple forms of G4-amylase from* Pseudomonas stutzeri* strains have been reported. Robyt and Ackerman [19] reported that* P. stutzeri* NRRL B-3389 produced seven G4-amylases which differed in molecular mass and isoelectric point. Strain MS300 was found to produce two major G4-amylases (amylases A and B) and two minor amylases (amylases C and D) [23]. However, the G4-amylase of* P. stutzeri* MO-19 has been found to exist in two forms. One of these is a 57 kDa protein (G4-1); the other is a 46 kDa protein (G4-2) which was derived by the limited proteolysis of G4-l [20].

### 3.2. Biochemical Characterization of Crude *α*-Amylase Preparation

#### 3.2.1. Effect of Temperature on *α*-Amylase Activity and Stability

**Figure 4:**
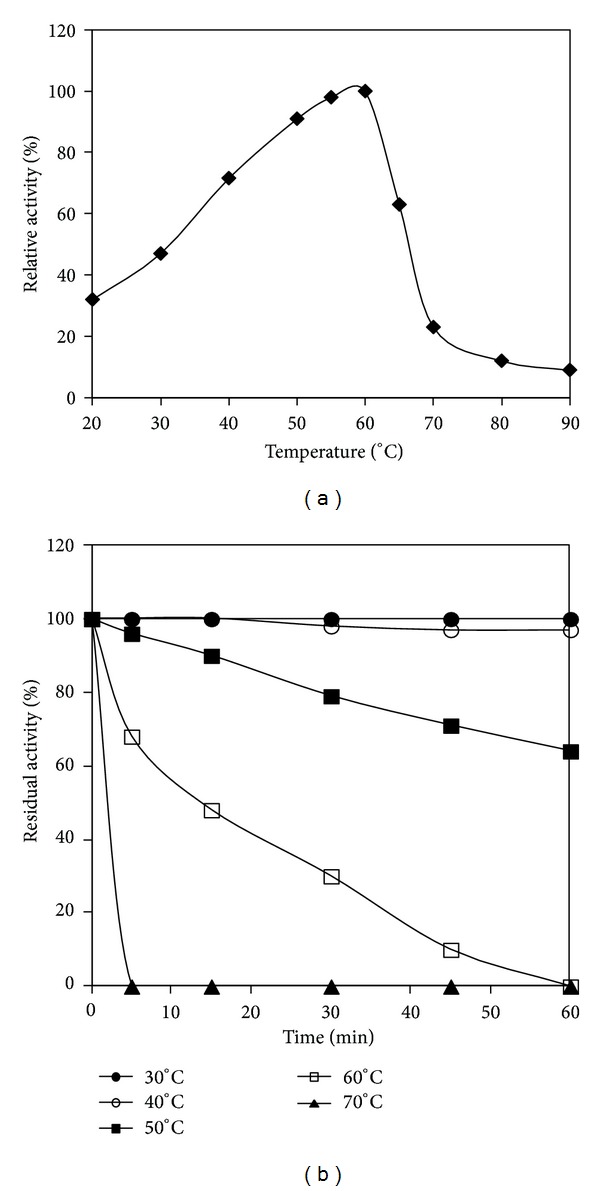
Temperature profile (a) and thermal stability (b) of* P. stutzeri* crude enzyme. Amylolytic activity was assayed at different temperatures ranging from 20 to 90°C at pH 8.0. The activity of the enzyme at 60°C was taken as 100%. To assess the thermostability, the* P. stutzeri* crude enzyme was heated at the indicated temperatures. The residual activity was assayed at pH 8.0 and 60°C. The nonheated crude enzyme was considered as the control (100%).

The temperature activity profile shows that the crude *α*-amylase was active at temperatures ranging from 20 to 70°C with an optimum activity at 60°C (Figure 4(a)). The relative activities at 65 and 70°C were about 63% and 23%, respectively. At 80°C, only 12% of the optimum activity was detected. While the optimum activity temperature of the crude *α*-amylase from* P. stutzeri* is comparable to that of the G4-*α*-amylase amyl I from* Bacillus* GM8901 [32], it was higher than that of previously reported maltotetraose-forming amylases from* Pseudomonas* species such as* Pseudomonas* MS300 [23] and* Pseudomonas stutzeri *NRRL B-3389 [33], which were reported to have optima temperatures of 40 and 45°C, respectively.

The effect of temperature on *α*-amylase stability was determined by incubating the enzyme preparation at different temperatures ranging from 30 to 70°C (Figure 4(b)). The crude amylase of the AS22 strain was stable at temperatures below 40°C. However, the activity decreased above 40°C and retained about 64% of its initial activity after 1 hour of incubation at 50°C. After 30 min of preincubation at 60°C, AS22 amylolytic preparation retained only 30% of its initial activity, showing therefore better thermostability than G4-amylases A and B from* Pseudomonas *MS300, which were reported to loose completely their activities after heating at 60°C for 30 min [23].

#### 3.2.2. Effect of pH on *α*-Amylase Activity and Stability

**Figure 5:**
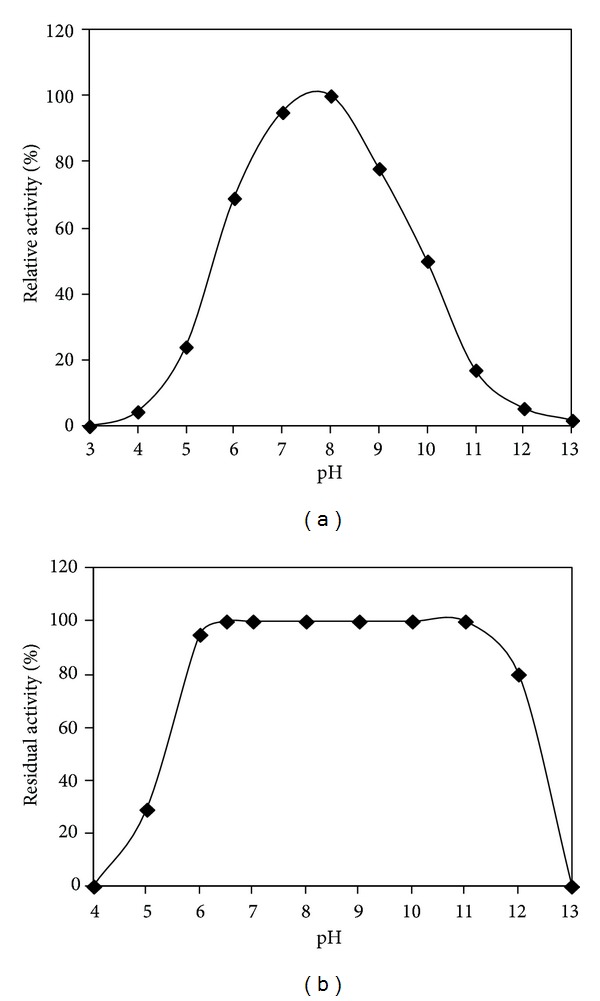
pH activity (a) and pH stability (b) of* P. stutzeri* crude enzyme. Amylolytic activity was assayed in the pH range of 3.0 to 13.0 at 60°C. The maximum activity obtained at pH 8.0 was considered as 100% activity. To assess the pH stability, the* P. stutzeri* crude enzyme was preincubated at the indicated pH at 30°C for 60 min and the residual enzyme activity was determined at pH 8.0 and 60°C. The activity of the crude enzyme before incubation was taken as 100%. Buffer solutions used for pH activity and stability are presented in Materials and Methods.

The pH activity profile reported in Figure 5(a) showed that the crude enzyme was active between pH 5.0 and pH 11.0 with an optimum at around pH 8.0. The relative activities at pH 6.0 and pH 10.0 were approximately 69% and 50% of that at pH 8.0, respectively. However, the activity decreased significantly to 20% at pH 5.0 and pH 11.0. Most of the earlier studies on* Pseudomonas* maltotetraose-forming amylases revealed an optimum pH range between 6.0 and 7.0 [20, 22, 33].

The crude *α*-amylase was completely stable in the pH range 6.0–11.0 since it retained its full activity after 1 hour of incubation at 30°C (Figure 5(b)) and retained up to 80% of its original activity at pH 12.0. However, at pH 5.0 the amylase activity decreased by 80% of the maximum activity at pH 8.0 and was completely lost at pH 4.0. According to previous studies, the crude *α*-amylase from* P. stutzeri *AS22 was found to be as stable as the purified G4-amylase from* P. stutzeri* NRRLB-3389 towards the same pH range but interestingly higher than G4-amylases from* Pseudomonas* sp. MS300 and* P. stutzeri* MO-19 which were stable in the pH range of 7.0~9.0 and 6.5~9.5, respectively [20, 23, 33].

#### 3.2.3. Effect of Metal Ions, Surfactants, and Enzyme Inhibitors

Because most of *α*-amylases are known to be metal ion-dependent enzymes [9], the effect of metal ions on the crude *α*-amylase activity was measured in the presence of various metal ions at a concentration of 5 mM. The enzyme activities were stimulated in the presence of Ca^2+^ and Ba^2+^ ions by 123% and 110%, respectively (data not shown). On the other hand, a strong inhibitory effect was observed in the presence of Hg^2+^, Zn^2+^, Mn^2+^, and Cd^2+^ and more than 90% of the amylolytic activity was lost. Results suggest that AS22 amylolytic preparation did not require any metal ions for catalytic activity except Ca^2+^ and Ba^2+^. According to Gupta et al. [18] and Sharma and Satyanarayana [34], *α*-amylase contains at least one Ca^2+^ ion and affinity of Ca is much stronger than that of other metal ions.

The stability of the AS22 crude *α*-amylase was also studied by incubating the amylolytic preparation in the presence of some enzyme inhibitors and surfactants for 30 min at room temperature (Table 5). Among all inhibitors tested, the chelating agent EDTA and the reducing agent *β*-mercaptoethanol inactivated the amylolytic activity where it could retain 70% and 66% of its initial activity, respectively. However, the serine proteinase inhibitor PMSF showed no significant inhibition (10%).

The amylolytic preparation revealed a high stability in the presence of 5% of the nonionic surfactants (Tween 20, Tween 80, and Triton X-100) retaining more than 80% residual activity and was fully stable in the presence of 0.1% SDS. However, with further increase in SDS concentration to 1%, around 50% loss of amylolytic activity was observed.

#### 3.2.4. Action Pattern of the Amylolytic Preparation

**Figure 6:**
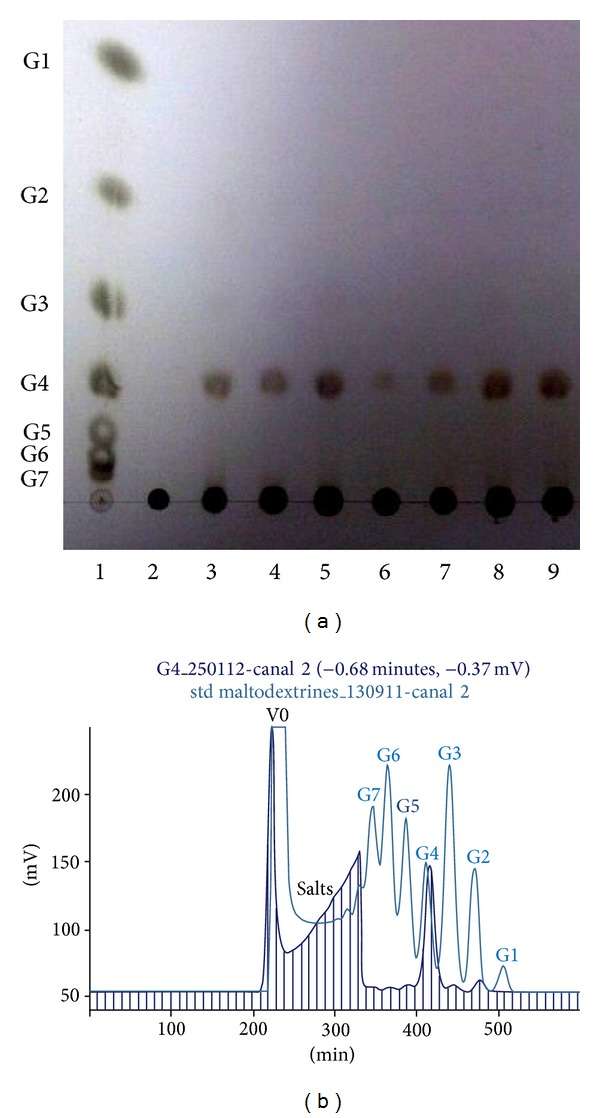
Thin-layer chromatography (a) and GPC (b) analysis of the soluble starch hydrolysates by crude *α*-amylases of* P. stutzeri* AS22. The reaction mixture containing 0.2 U of *α*-amylase activity and 1% substrate in 0.1 M Tris-HCl buffer (pH 8.0) was incubated at 60°C. (a) Lane 1: standard maltooligosaccharides (G1–G7), lane 2: starch used as substrate, and lanes 3–9: soluble starch hydrolyzed by* P. stutzeri* AS22 after 2 min, 10 min, 30 min, 1 h, 2 h, 4 h, and 6 h. (b) End products analysis after 6 h hydrolysis of soluble starch. GPC analysis was performed on a Bio-Gel P2 column (1.5 × 200 cm) eluted with water at a rate of 30 mL/h. The different oligosaccharides released after starch hydrolysis were fractionated and quantified in comparison with standard oligosaccharides ranging from G1 to G7. G1: glucose, G2: maltose, G3: maltotriose, G4: maltotetraose, G5: maltopentaose, G6: maltohexaose, and G7: maltoheptaose. V0: the void volume.

To examine the mode of action of* P. stutzeri* AS22 crude enzyme on starch hydrolysis, starch was treated with the culture supernatant and the reaction products of samples taken at different time intervals were analysed by TLC. As shown in Figure 6(a), at an early stage of hydrolysis (2 min of reaction), maltotetraose (G4) was released without any intermediate as the specific end product.

On the basis of its mode of action,* P. stutzeri* amylolytic preparation consists in a maltotetraose-forming-*α*-amylase activity. Previously, maltotetraose-forming-amylases were mainly discovered in* Pseudomonas* strains such as* Pseudomonas stutzeri* [19, 20, 33, 35]* Pseudomonas saccharophila* [24],* Pseudomonas *sp. IMD 353 [22], and* Pseudomonas* MS300 [23] and also in* Bacillus* strains such as* Bacillus *sp. GM8901 [32] and* Bacillus halodurans* MS-2-5 [36].

### 3.3. Course of Hydrolysis of Starch with the Amylolytic Preparation

To quantify the amounts of the products generated from starch hydrolysis by* P. stutzeri* AS22 crude enzyme, the hydrolysates were analyzed at function of time by gel permeation chromatography (GPC) which was carried out as written in Materials and Methods.

Table 6 and Figure 6(b) summarize the GPC analysis of maltooligosaccharides formed at various time periods of hydrolysis reaction. It is very clear that maltotetraose (G4) is the specific product formed with a high degree of purity in the hydrolysates even from the early stage of the reaction (5 min). While traces of contaminating maltose, maltotriose, maltopentaose and maltohexaose could be produced, glucose was not detectable. Moreover, maltotetraose concentration remains constant at 97–100% from 5 min to 6 h of hydrolysis reaction. This clearly indicates that initially formed maltotetraose was not hydrolyzed and therefore the* P. stutzeri* amylolytic preparation consists principally of a maltotetraose-forming amylase.

Interestingly, the maltotetraose production reached its maximum of 5.3 g/L after 15 min of hydrolysis with negligible amounts of maltose (0.06 g/L), maltotriose (0.03 g/L), and maltopentaose (0.02 g/L). In addition, more than half (55%) of the starch hydrolysis was achieved in 15 min without optimization. Therefore, optimization of reaction conditions especially starch concentration and amylase amount may further increase its yield.

Despite the fact that the starch hydrolysis reaction by* P. stutzeri* crude enzyme occurred without optimization, the level of maltotetraose produced was higher as compared with that of the known maltotetraose-forming amylases. Interestingly,* P. stutzeri* AS22 amylase preparation produced maltotetraose representing up to 97% as compared with the yield of maltotetraose released by the action of* P. stutzeri* [37] purified amylase which was estimated to be only 55%. The isolated amylase from* Bacillus circulans* MG-4 [38] yields 64.9% of maltotetraose with amounts of contaminating glucose, maltose, and maltotriose. Therefore, besides the cost effectiveness offered by its use, the amylolytic preparation of* P. stutzeri* AS22 seems to be quite different, with respect to its efficiency, from the other G4-amylases from* Pseudomonas* species.

## 4. Conclusion

The nature of culture conditions and composition of media for optimal production of *α*-amylase by* P. stutzeri *AS22, as well as its crude enzyme biochemical characterization, have been developed in this study.

Incubation period of 24 h, initial pH of 8.0, 30°C incubation temperature, and an agitation speed of 200 rpm were found to be optimum for the production of *α*-amylase. Amylase production reached 6.8 U/mL by supplementing the fermentation media with starch (1%), yeast extract (0.5%), MgSO_4_ (0.01%), NaCl (0.05%), K_2_HPO_4_ (0.14%), KH_2_PO_4_ (0.07%), and CaCl_2_ (0.04%).

Temperature 60°C and pH 8.0 were found to be the best for amylase activity. The crude *α*-amylase was highly stable at the pH ranging from 6.0 to 12.0 and amylase activity was enhanced in presence of Ba^2+^ and Ca^2+^ ions.

The production of high yields of a specific maltooligosaccharide on degradation of starch by *α*-amylases is of considerable commercial interest. An important characteristic of the* P. stutzeri *AS22 amylase preparation is that it hydrolyses potato starch (1%) into high amount of maltotetraose (5.3 g/L) without synchronous glucose production. Interestingly, this amylase preparation, producing maltotetraose on a higher yield (98%) as compared with that of previously reported G4-amylases, seems to be promising in the manufacture of high maltotetraose syrups from starch.

## Figures and Tables

**Table 1:** Effects of different carbon sources on the production of *α*-amylase by *P. stutzeri* AS22.

Carbon sources	Final pH value	Biomass (OD_600_)	*α*-Amylase activity (U/mL)
Lactose	7.49	0.1	0.055
Glucose	5.69	0.8	0.1
Maltose	6.62	1.0	0.5
Potato starch	6.52	0. 8	0.8
Wheat starch	6.69	1.04	0.1
Maize starch	6.59	0.5	0.17

Cultivation was performed for 24 h at 37°C with shaking at 200 rpm in media that consisted of (g/L) carbon source 10, ammonium sulphate 1, K_2_HPO_4_ 1.4, KH_2_PO_4_ 0.7, MgSO_4_ 0.1, and NaCl 0.5 and that were inoculated at initial OD of 0.016 and adjusted to pH 8.0.

**Table 2:** Effects of different nitrogen sources supplemented to the potato starch on the production of *α*-amylase by *P. stutzeri* AS22.

Nitrogen sources	Final pH value	Biomass (OD_600_)	*α*-Amylase activity (U/mL)
NH_4_Cl	6.6	0.94	0.5
(NH_4_)_2_SO_4_	6.5	0.9	0.7
Soya peptone	7.9	3.21	0.97
Casein	7.85	1.61	1.0
Pastone	7.78	3.08	1.5
Yeast extract	7.87	3.56	2.5

Cultivation was performed for 24 h at 37°C with shaking at 200 rpm in media that consisted of (g/L) potato starch 10, K_2_HPO_4_ 1.4, KH_2_PO_4_ 0.7, MgSO_4_ 0.1, NaCl 0.5, and different nitrogen sources (1 g/L) and were adjusted to pH 8.0.

**Table 3:** Effect of different initial pH values of fermentation medium on the production of *α*-amylase by *P. stutzeri* AS22.

Initial pH value	Final pH value	Biomass (OD_600_)	*α*-Amylase activity (U/mL)
6.0	4.73	3.9	0
7.0	7.2	4.9	3.8
8.0	8.0	5.6	5.9
9.0	8.55	5.5	5.85
10.0	8.9	5.5	5.8
11.0	9.22	5.6	5.6
12.0	9.32	5.4	5.4

Cultivation was performed for 24 h at 30°C with shaking at 200 rpm in media that consisted of (g/L) potato starch 10, yeast extract 5, K_2_HPO_4_ 1.4, KH_2_PO_4_ 0.7, MgSO_4_ 0.1, and NaCl 0.5 and were adjusted to different initial pH values.

**Table 4:** Effect of supplementation of the culture medium with various salts on the production of *α*-amylase by *P. stutzeri* AS22.

Chemicals	Concentration (g/L)	Final pH value	Biomass (OD_600_)	*α*-Amylase activity (U/mL)
None		7.2	4.77	3.5
Control		7.8	5.45	5.9
NaCl	0.5	7.32	4.9	3.8
K_2_HPO_4_	1.4	7.6	2.57	5.19
KH_2_PO_4_	0.7	7.7	2.01	5.9

MgSO_4_	0.1	7.08	5.43	4.13
	0.4	7.0	6.35	4.42
	0.8	7.3	5.73	3.24

CaCl_2_	0.1	6.9	7.15	6.01
	0.4	6.7	6.3	6.6
	0.8	6.5	6.19	6.5
	1.2	6.3	6.2	4.6

Control + CaCl_2_ (0.4 g/L)		7.2	6.2	6.8

Cultivation was performed for 24 h at 30°C with shaking at 200 rpm in media that consisted of (g/L) potato starch 10, yeast extract 5, and different salts and were adjusted to pH 8.0. Control: potato starch 10, yeast extract 5, K_2_HPO_4_ 1.4, KH_2_PO_4_ 0.7, MgSO_4_ 0.1, and NaCl 0.5 g/L.

**Table 5:** Stability of the crude *α*-amylase in the presence of various additives.

Additives	Concentration	Remaining activity (%)
None	—	100
Inhibitors		
PMSF	5 mM	90
*β*-Mercaptoethanol	5 mM	66
EDTA	5 mM	70
Surfactants		
Tween 20	5% (v/v)	81
Tween 80	5% (v/v)	90
Triton X-100	5% (v/v)	82
SDS	0.1% (w/v)	100
	1% (w/v)	49

Enzyme activity measured in the absence of any additive was taken as 100%. The remaining *α*-amylase activity was measured after preincubation of the crude *α*-amylase with each additive at room temperature for 30 min.

**Table 6:** Time course of maltooligosaccharides production from 1% soluble starch by *P. stutzeri* crude enzyme.

Hydrolysis time	Oligosaccharides concentration (g/L) and **percentage (%)**					
	G1	G2	G3	G4	G5	G6
0 min	0	0	0	0	0	0
5 min	0	0	0	2.68 **(**100**)**	0	0
15 min	0	0.06 **(1.1)**	0.03 **(0.5)**	5.31 **(**98**)**	0.02 **(0.4)**	0
30 min	0	0.08 **(1.6)**	0.05 **(0.95)**	5.1 **(**97**)**	0.023 **(0.45)**	0
1 h	0	0.04 **(0.8)**	0.03 **(0.8)**	4.9 **(**98**)**	0.02 **(0.4)**	0.01 **(0.2)**
2 h	0	0.058 **(1.2)**	0.03 **(0.6)**	4.75 **(97.5)**	0.02 **(0.41)**	0.014 **(0.29)**
6 h	0	0.07 **(1.4)**	0.05 **(**1**)**	4.92 **(**97**)**	0.04 **(0.8)**	0.02 **(0.4)**

0.1 unit of the *α*-amylase activity was reacted with 1% of soluble starch at 60°C and pH 8.0 and reaction products were analyzed at different time intervals.

## References

[1] Fujita M, Torigoe K, Nakada T (1989). Cloning and nucleotide sequence of the gene (*amy*P) for maltotetraose-forming amylase from *Pseudomonas stutzeri* MO-19. *Journal of Bacteriology*.

[2] Barreteau H, Delattre C, Michaud P (2006). Production of oligosaccharides as promising new food additive generation. *Food Technology and Biotechnology*.

[3] Nakakuki T (2002). Present status and future of functional oligosaccharide development in Japan. *Pure and Applied Chemistry*.

[4] Palacios HR, Schwarz PB, D’Appolonia BL (2004). Effect of *α*-amylases from different sources on the retrogradation and recrystallization of concentrated wheat starch gels: relationship to bread staling. *Journal of Agricultural and Food Chemistry*.

[5] Rivero-Urgell M, Santamaria-Orleans A (2001). Oligosaccharides: application in infant food. *Early Human Development*.

[6] Aiyer PV (2005). Amylases and their applications. *African Journal of Biotechnology*.

[7] Malabendu J, Chiranjit M, Saptadip S (2013). Salt-independent thermophilic *α*-amylase from *Bacillus megaterium* VUMB109: an efficacy testing for preparation of maltooligosaccharides. *Industrial Crops and Products*.

[8] Duedahl-Olesen L, Matthias Kragh K, Zimmermann W (2000). Purification and characterisation of a malto-oligosaccharide-forming amylase active at high pH from *Bacillus clausii* BT-21. *Carbohydrate Research*.

[9] Pandey A, Nigam P, Soccol CR, Soccol VT, Singh D, Mohan R (2000). Advances in microbial amylases. *Biotechnology and Applied Biochemistry*.

[10] Lalucat J, Bennasar A, Bosch R, García-Valdés E, Palleroni NJ (2006). Biology of *Pseudomonas stutzeri*. *Microbiology and Molecular Biology Reviews*.

[11] Maalej H, Hmidet N, Ghorbel-Bellaaj O, Nasri M (2013). Purification and biochemical characterization of a detergent stable *α*-amylase from *Pseudomonas stutzeri* AS22. *Biotechnology and Bioprocess Engineering*.

[12] Miller JH (1972). *Experiments in Molecular Genetics*.

[13] Miller GL (1959). Use of dinitrosalicyIic acid reagent for determination of reducing sugar. *Analytical Chemistry*.

[14] Sharma A, Satyanarayana T (2011). Optimization of medium components and cultural variables for enhanced production of acidic high maltose-forming and Ca^2+^-independent *α*-amylase by *Bacillus acidicola*. *Journal of Bioscience and Bioengineering*.

[15] Babu KR, Satyanarayana T (1993). Parametric optimization of extracellular *α*-amylase production by thermophilic *Bacillus coagulans*. *Folia Microbiologica*.

[16] Tonomura K, Suzuki H, Nakamura N, Kuraya K, Tanabe O (1961). On the inducers of *α*-amylase formation in *Aspergillus oryzae*. *Agricultural and Biological Chemistry*.

[17] Morkeberg R, Carlsen M, Nielsen J (1995). Induction and repression of *α*-amylase production in batch and continuous cultures of *Aspergillus oryrae*. *Microbiology*.

[18] Gupta R, Gigras P, Mohapatra H, Goswami VK, Chauhan B (2003). Microbial *α*-amylases: a biotechnological perspective. *Process Biochemistry*.

[19] Robyt JF, Ackerman RJ (1971). Isolation, purification, and characterization of a maltotetraose-producing amylase from *Pseudomonas stutzeri*. *Archives of Biochemistry and Biophysics*.

[20] Nakada T, Kubota M, Sakai S, Tsujisaka Y (1990). Purification and characterization of two forms of maltotetraose-forming amylase from *Pseudomonas stutzeri*. *Agricultural and biological chemistry*.

[21] Fogarty WM, Kelly CT (1990). *Industrial Enzymes and Biotechnology*.

[22] Fogarty WM, Kelly CT, Bourke AC, Doyle EM (1994). Extracellular maltotetraose-forming amylase of *Pseudomonas* sp. IMD 353. *Biotechnology Letters*.

[23] Kobayashi H, Takaki Y, Kobata K, Takami H, Inoue A (2000). Characterization of *α*-maltotetraohydrolase produced by *Pseudomonas* sp. MS300 isolated from the deepest site of the Mariana trench. *JAMSTEC Journal of Deep Sea Research*.

[24] Kobayashi S, Okemoto H, Hara K, Hashimoto H, Yamasato K (1991). Preparation and some properties of a novel maltotetraose-forming enzyme of *Pseudomonas saccharophila*. *Journal of the Japanese Society of Starch Science*.

[25] Kathiresan K, Manivannan S (2006). *α*-Amylase production by *Penicillium fellutanum* isolated from mangrove rhizosphere soil. *African Journal of Biotechnology*.

[26] Rameshkumar A, Sivasudha T (2011). Optimization of nutritional constitute for enhanced alpha amylase production using by solid state fermentation technology. *International Journal of Microbiological Research*.

[27] Sivaramakrishnan S, Gangadharan D, Nampoothiri KM, Soccol CR, Pandey A (2006). *α*-amylases from microbial sources—an overview on recent developments. *Food Technology and Biotechnology*.

[28] Malhotra R, Noorwez SM, Satyanarayana T (2000). Production and partial characterization of thermostable and calcium-independent *α*-amylase of an extreme thermophile *Bacillus thermooleovorans* NP54. *Letters in Applied Microbiology*.

[29] Subramanian G, Ayyadurai S, Sharma T, Singh SA, Rele M, Kumar LS (2012). Studies on maltoheaose (G6) producing alkaline amylase from a novel alkalophilic *Streptomyces* species. *The IIOAB Journal*.

[30] Konsoula Z, Liakopoulou-Kyriakides M (2007). Co-production of *α*-amylase and *β*-galactosidase by *Bacillus subtilis* in complex organic substrates. *Bioresource Technology*.

[31] Sudha (2012). Effect of different concentrations of metal ions on alpha amylase production by *Bacillus amyloliquefaciens*. *Research in Biotechnology*.

[32] Kim TU, Gu BG, Jeong JY, Byun SM, Shin YC (1995). Purification and characterization of a maltotetraose-forming alkaline *α*- amylase from an alkalophilic *Bacillus* strain, GM8901. *Applied and Environmental Microbiology*.

[33] Sakano Y, Kashiwagi Y, Kobayashi T (1982). Purification and properties of an exo-*α*-amylase from *Pseudomonas stutzeri*. *Agricultural and Biological Chemistry*.

[34] Sharma A, Satyanarayana T (2013). Microbial acid-stable *α*-amylases: characteristics, genetic engineering and applications. *Process Biochemistry*.

[35] Lee MJ, Chung MJ (1984). Studies on the exo-maltotetraohydrolase of *Pseudomonas stutzeri* IAM 12097—part II: characteristics of exo-maltotetraohydrolase. *Journal of the Korean Chemical Society*.

[36] Murakami S, Nagasaki K, Nishimoto H (2008). Purification and characterization of five alkaline, thermotolerant, and maltotetraose-producing *α*-amylases from *Bacillus halodurans* MS-2-5, and production of recombinant enzymes in *Escherichia coli*. *Enzyme and Microbial Technology*.

[37] Schmidt J, John M (1979). Starch metabolism in *Pseudomonas stutzeri*. I. Studies on maltotetraose-forming amylase. *Biochimica et Biophysica Acta*.

[38] Takasaki Y, Shinohara H, Tsuruhisa M, Hayashi S, Imada K (1991). Maltotetraose-producing amylase from *Bacillus* sp. MG-4. *Agricultural and Biological Chemistry*.

